# Strukturelle, prozedurale und personelle Voraussetzung für die Erbringung radioonkologischer und strahlentherapeutischer Leistungen 2023 in Deutschland – ein Positionspapier der Deutschen Gesellschaft für Radioonkologie (DEGRO)

**DOI:** 10.1007/s00066-023-02105-6

**Published:** 2023-06-19

**Authors:** Rainer Fietkau, Ulrike Höller, Mechthild Krause, Cordula Petersen, Michael van Kampen, Dirk Vordermark, Jochen Willner

**Affiliations:** 1 Strahlenklinik, Friedrich-Alexander-Universität Erlangen-Nürnberg, Erlangen, Deutschland Universitätsstr. 27, 91054; 2 Deutsche Gesellschaft für Radioonkologie (DEGRO), Berlin, Deutschland; 3 Klinik und Poliklinik für Strahlentherapie und Radioonkologie, Universitätsklinikum Carl Gustav Carus, Technische Universität Dresden, Dresden, Deutschland; 4 Klinik für Strahlentherapie und Radioonkologie, Universitätsklinikum Hamburg-Eppendorf, Martinistraße 52, 20246 Hamburg, Deutschland; 5 Ambulanzzentrum, Universitätsklinikum Hamburg-Eppendorf, Martinistraße 52, 20246 Hamburg, Deutschland; 6 Radioonkologische Klinik, Krankenhaus Nordwest, Frankfurt/Main, Deutschland; 7 Klinik für Strahlentherapie, Universitätsklinikum Halle (Saale), Halle (Saale), Deutschland; 8 Klinik für Strahlentherapie, Klinikum Bayreuth GmbH, Medizincampus Oberfranken der FAU Erlangen, Bayreuth, Deutschland

## Einleitung

Die Regierungskommission hat ihren Vorschlag zur Krankenhausreform vorgestellt, die das Ziel der optimalen Ressourcennutzung hat. In diesem Prozess muss die Radioonkologie/Strahlentherapie, eine der drei tragenden Säulen der Krebsbehandlung und eigenes Fachgebiet, adäquat berücksichtigt werden. Die Deutsche Gesellschaft für Radioonkologie (DEGRO) beschreibt die strukturellen und prozeduralen Voraussetzungen für eine patientengerechte Einbindung der Radioonkologie vor dem Hintergrund der aktuellen Entwicklungen der Radioonkologie in Deutschland [1].

## Geplante Krankenhausreform

Ziel der geplanten Krankenhausreform ist es, die in § 1 des Krankenhausfinanzierungsgesetzes geforderte „qualitativ hochwertige, patienten- und bedarfsgerechte Versorgung der Bevölkerung mit leistungsfähigen, qualitativ hochwertig und eigenverantwortlich wirtschaftenden Krankenhäusern“ landesweit zu realisieren. Eine Regierungskommission aus 17 Experten verschiedenster Fachdisziplinen erarbeitete Vorschläge für eine moderne und bedarfsgerechte Krankenhausversorgung. In ihrer Stellungnahme für die grundlegende Reform der Krankenhausvergütung schlägt sie drei Kernbestandteile vor:

„1. eine einheitliche Definition von Krankenhaus-Versorgungsstufen (Leveln), um lokale, regionale und überregionale Versorgungsaufträge abzugrenzen

2. ein System von Leistungsgruppen, die passgenauer als durch Fallpauschalen/DRGs (hohe Granularität) und Fachabteilungen (niedrige Spezifität) den Leveln zugeordnet und dem Bevölkerungsbedarf angepasst werden können

3. Reduktion der mengenbezogenen Komponente zugunsten einer bedarfs-gerechten und qualitätsorientierten Vorhaltefinanzierung“ [2].

Damit soll die Finanzierung allein durch Fallpauschalen abgelöst werden, um die Strukturqualität der Krankenhausversorgung zu verbessern und zu sichern.

Die Regierungskommission veröffentlichte eine Liste von Leistungsgruppen, die allerdings noch nicht vollständig ist. Daher haben die onkologisch tätigen Fachgebiete in einem von der AWMF geführten Prozess einen Umsetzungsvorschlag erarbeitet, der die Onkologie entsprechend ihrer quantitativen und ökonomischen Bedeutung vollständig abbildet [3]. Er fokussiert entlang des Patientenpfads auf die Kooperationen zwischen den Fachdisziplinen, Berufsgruppen und den Krankenhäusern bzw. ambulanten Strukturen wie Arztpraxen oder medizinischen Versorgungszentren (MVZ).

Im Entwurf der Regierungskommission wurde die Radioonkologie bisher noch nicht als Leistungsbereich definiert. Ergänzend zu der Stellungnahme der onkologischen Fachgesellschaften in der AWMF wird daher im Folgenden der Leistungsbereich Radioonkologie in Bezug auf die erforderliche Strukturqualität und die Versorgungslevel beschrieben.

## Stellung der Radioonkologie in der Krebstherapie

Das Fachgebiet Radioonkologie und Strahlentherapie hat mit der Gründung der eigenen Fachgesellschaft DEGRO (Deutsche Gesellschaft für Radioonkologie) 1995 in Deutschland eine enorme Entwicklung genommen. Dem voraus ging ein rasanter internationaler Aufschwung neuer technischer, klinischer (z. B. Kombination von Radio- und Chemotherapie) und biologischer Möglichkeiten der kurativen Strahlentherapie. Die wissenschaftliche und klinische Entwicklung führte zur Etablierung als eigenständiges Fachgebiet. Heute stellt die Radioonkologie neben Systemtherapie und Chirurgie eine der zentralen Säulen in der interdisziplinären Onkologie dar. Die Radioonkologie ist ein essenzieller Pfeiler in jedem Comprehensive Cancer Center (CCC) und gleichermaßen in den zertifizierten onkologischen Zentren und den Organkrebszentren.

Neben der technischen Verbesserung der Behandlungsgeräte (in der Regel Linearbeschleuniger) und der Brachytherapie setzen sich in der kurativen Onkologie zunehmend multimodale Behandlungskonzepte in Form von Radio-Chemo‑/Immuntherapien oder Kombinationen mit einer Operation in verschiedensten zeitlichen Sequenzen durch. Diese kombinierten Therapiekonzepte stellen zunehmend hohe Anforderungen an die Radioonkologie in der interdisziplinären Zusammenarbeit und an die technischen Vorhaltungen.

Insgesamt erhalten etwa 50 % aller Krebspatientinnen und -patienten im Laufe ihrer Krankheit eine Strahlentherapie, häufig kombiniert mit einer medikamentösen Tumortherapie [4].

## Radioonkologie als Beispiel der sektorenübergreifenden Therapie

Im Fachbereich Radioonkologie erfolgt bereits heute die Mehrzahl der Bestrahlungen ambulant, nur ca. 15 % der Bestrahlungsleistungen werden unter stationären Bedingungen appliziert [1]. Hierbei sinkt oft der stationäre Anteil einer mehrwöchigen Gesamtbehandlung, die bereits heute überwiegend ambulant erfolgt.

Die Analyse der diagnosebezogenen Gruppenstatistik (G-DRG-Statistik) in Deutschland des Forschungsdatenzentrums des Statistischen Bundesamts und der ambulanten Abrechnungsdaten ergab für den Zeitraum von 2015 bis 2018 eine Reduktion aller stationären Bestrahlungsleistungen von 3 % sowie eine Zunahme der ambulanten Bestrahlungen von Tumorpatienten um 7 %. Damit dürfte das Ambulantisierungspotenzial der Strahlentherapie/Radioonkologie zu großen Teilen bereits ausgenutzt sein.

Eine Besonderheit der radioonkologischen Behandlung ist die Notwendigkeit, unter allen Umständen Therapiepausen oder Verzögerungen der Therapie zu verhindern, da diese nachweislich zu einer Verschlechterung der Therapieergebnisse führen [5]. Eine weitere Besonderheit der Radioonkologie ist die aus technischen Gründen nötige Bindung der Patient:innen an das initial festgelegte Bestrahlungsgerät und die initial betreuende radioonkologische Einheit. Jeder Wechsel zum Beispiel zwischen verschiedenen Einrichtungen führt zur Notwendigkeit der medizinischen und physikalischen Neuplanung der Therapie und damit neben dem hohen Einsatz von Ressourcen zwangsläufig zu einer mehrtägigen Therapieunterbrechung und ist somit zu vermeiden. Dies erzwang schon frühzeitig die enge Verknüpfung der ambulanten und stationären Therapie.

Für bestimmte Patientengruppen und für bestimmte Therapieverfahren muss die Therapie mindestens partiell stationär durchgeführt werden oder die Option einer stationären Therapie vorgehalten werden. Die patientenbedingten Gründe für eine (partiell) stationäre Durchführung der Therapie sind vielfältig: sehr weit fortgeschrittene Tumorerkrankung (eingeschränkte Belastbarkeit der Patient:innen), Schmerzbestrahlung bei ausgedehnter Metastasierung mit simultan erforderlicher Einstellung der Schmerzmedikation, reduzierter Allgemein- und Ernährungszustand (Notwendigkeit der bilanzierten parenteralen Ernährung) oder Einschränkung der kognitiven Funktionen (z. B. Patient:innen mit Hirntumoren oder Hirnmetastasen).

Einige Verfahren der Radioonkologie, wie zum Beispiel die interstitielle Brachytherapie (gynäkologische Tumoren, Prostatakarzinom, Mammakarzinom und Rezidivbestrahlungen) und die Ganzkörperbestrahlung (im Rahmen einer Knochenmarktransplantation), lassen sich wegen der nötigen Begleitmaßnahmen (Narkose, Supportivtherapie etc.) nur unter stationären Bedingungen durchführen. Ebenso sind bestimmte Verfahren der medikamentösen Tumortherapie in Kombination mit Bestrahlung wegen der nötigen zeitlichen und örtlichen Synchronisierung und/oder des Überwachungsbedarfs nur unter stationären Bedingungen durchführbar. Die Patient:innen erhalten typischerweise eine Chemotherapie an wenigen Tagen im drei- oder vierwöchentlichen Rhythmus simultan zur mehrwöchigen Strahlentherapie. Sie werden während des stationären Aufenthalts und in den Intervallen weiter ambulant durch die initiale Einrichtung (z. B. angegliedertes MVZ, Kooperation mit Praxis) durchgehend am gleichen oder baugleichen Gerät bestrahlt.

Kombinationstherapien erfordern eine Vorhaltung von stationären Betten für ambulante Patient:innen, sodass sie bei einer Verschlechterung des Allgemeinzustands oder bei Komplikationen unter laufender Therapie (z. B. Sepsis, eingeschränkte Hämatopoese, massive Durchfälle mit Elektrolytverschiebungen) umgehend stationär aufgenommen und ohne Verzögerung weiter radioonkologisch behandelt werden können. Sowohl die Systemtherapie als auch die Strahlentherapie und Supportivtherapie müssen individuell so adaptiert werden, dass das Behandlungsziel nicht gefährdet wird. Das erfordert radioonkologische Fachkompetenz mit Kenntnis der Strahlendosisverteilung, der Interaktion der Strahlen- und Systemtherapie und der Genese der Nebenwirkungen.

Zusammenfassend ist das Vorhalten einer stationären radioonkologischen Therapieoption die Grundvoraussetzung für den hohen Ambulantisierungsgrad der Radioonkologie.

## Anforderungen an die Radioonkologie im stationären Setting

An die Struktur- und Prozessqualität der stationären Radioonkologie werden hohe Anforderungen gestellt, um Systemtherapien, ungeplante, dringliche Supportivtherapien sowie Notfallbestrahlungen, z. B. bei beginnender Querschnittslähmung durch Tumorkompression am Rückenmark, Tumorblutung oder Blutgefäßkompression, sicherzustellen. Unter anderem müssen fachärztliche radioonkologische Kompetenz 24/7 und eine gut etablierte interdisziplinäre Zusammenarbeit mit anderen onkologischen Fachdisziplinen und Fachbereichen wie Schmerz- Physio‑, Palliativtherapie etc. verfügbar sein.

Die Ambulantisierung hat zur Umverteilung von komplexen Behandlungsfällen in Versorgungseinheiten, die stationäre und auch palliative und supportive Behandlungen anbieten können, und zur Konzentration betreuungsintensiver Patienten im stationären Bereich geführt. Diese Patienten haben üblicherweise eine messbar hohe Bewertungsrelation im Fallpauschalenkatalog oberhalb von 2.

**Figure 1:**
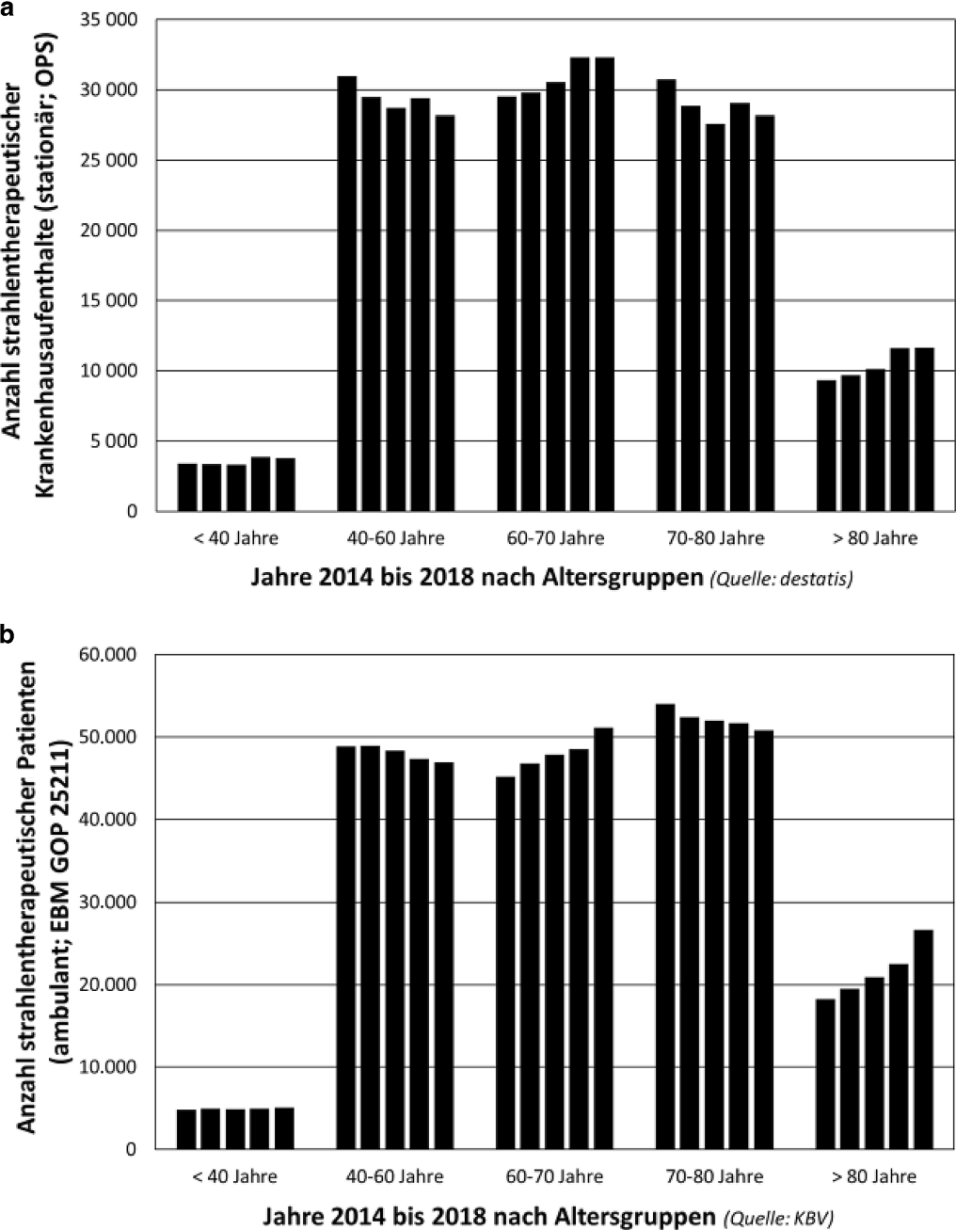

Vor allem aufgrund der demografischen Entwicklung ist zwischen 2015 und 2030 in Deutschland mit einem Anstieg der Krebsneuerkrankungen um rund 23 % zu rechnen [6]. Dadurch wird der Bedarf an strahlentherapeutischen Leistungen zur Versorgung von Tumorpatienten in den kommenden Jahren zunehmen. Zugleich steigt das Alter der Patienten – das mittlere Alter bei Krebsdiagnose in Deutschland betrug 2018 bereits 69 Jahre bei Frauen und 70 Jahre bei Männern [6]. Dies lässt einen überproportionalen Anstieg des Bedarfs an radioonkologischen Leistungen als nichtinvasive und risikoärmere Alternative zu einer operativen Therapie für die oft multimorbiden Patienten erwarten [7] und zeichnet sich ab (Abb. 1).

Insgesamt ergeben sich aus der steigenden Anzahl betagter, multimorbider Patienten, aus der Zunahme von komplexen Therapiekonzepten sowie aus der zunehmend langen Lebenserwartung von Krebspatienten auch mit fortgeschrittenen Krebsleiden erhöhte Anforderungen an die stationäre radioonkologische Patientenversorgung.

Diese Aufgaben in der Patientenversorgung und der Ausbildung von Nachwuchs im ärztlichen und technischen Dienst sind unterschiedlich für praxisbasierte, klinikbasierte sowie universitäre Einrichtungen. Vor allem klinikbasierte Einrichtungen haben die Aufgabe, in der ärztlichen Weiterbildung den Kompetenzerwerb in medikamentöser Tumortherapie, Supportivtherapie und Palliativtherapie komorbider und/oder betagter Patient: innen sowie im Management ausgeprägter Nebenwirkungen sicherzustellen [8].

## Vorschlag zur Definition Leistungsbereich Radioonkologie

Die Radioonkologie ist als Querschnittsfach Hauptkooperationspartner für alle onkologisch tätigen Fachbereiche und aufgrund ihrer Bedeutung für die Krebstherapie als Leistungsbereich zu definieren. Die Behandlung erfolgt dabei stationär, stationär-ambulant oder ambulant, immer aber in derselben Bestrahlungseinrichtung.

Entscheidend für gute klinische Ergebnisse und die Wirtschaftlichkeit der Therapie sind die Qualität des Netzwerks der onkologischen Fachdisziplinen, die Qualifikation und Expertise der einzelnen Behandelnden sowie die Qualität der Leistungserbringung selbst.

In der Radioonkologie werden grundsätzlich mit der Richtlinie zum Strahlenschutz in der Medizin die Anforderungen an Menge und Qualifikation der Behandelnden, an Maßnahmen zur Mitarbeitenden- und Patientensicherheit und an technische Mindestanforderungen zwingend vorgeschrieben [9]. Die Einhaltung der medizinischen und physikalischen Qualitäts- und Sicherheitsvorgaben wird in allen ambulanten und stationären Einrichtungen der Radioonkologie zweijährlich durch die Ärztlichen Stellen im Auftrag der Aufsichtsbehörden durch Expertenbegehung vor Ort anhand eines „Einheitlichen Bewertungssystems“ [10] überprüft.

Die Regierungskommission schlägt Versorgungslevel 2 und 3 für einzelne onkologische Leistungsgruppen, z. B. in der Dermatoonkologie, vor [2]. Dies wird von der DEGRO ebenso wie von allen anderen onkologisch tätigen Fachbereichen für sinnvoll gehalten und in einer entsprechenden Stellungnahme unter Führung der AWMF ausdrücklich begrüßt [3].

Unter anderem werden den Anforderungen an die Versorgungslevel die Anforderungen der Zertifizierung der Deutschen Krebsgesellschaft e. V. bzw. der Deutschen Krebshilfe zugrunde gelegt [11]. In diesem System der zertifizierten Zentren (Organkrebszentren, onkologischen Zentren und onkologischen Spitzenzentren) wurden die wesentlichen Anforderungen an eine qualitativ hochwertige Tumortherapie sowohl strukturell als auch prozedural organ- und fachspezifisch (Querschnittsfächer) erarbeitet. Der Erfolg des Konzepts konnte in der durch den Innovationsausschuss des Gemeinsamen Bundesausschusses geförderten Studie „Wirksamkeit der Versorgung in onkologischen Zentren“ (WiZen) nachgewiesen werden. Bei 10 von 11 untersuchten Tumorentitäten zeigten sich dabei signifikante Überlebensvorteile für die in zertifizierten Zentren behandelten Patient:innen bei gleichzeitig niedrigeren Behandlungskosten [12–14].

Eine Expertengruppe der DEGRO hat eine Matrix erarbeitet, die die Anforderungen an die Versorgungslevel 2 und 3 des Leistungsbereichs Radioonkologie definiert (Tab. 1). Ziel war es, die Kenngrößen einer „qualitativ hochwertigen, patienten- und bedarfsgerechten Versorgung der Bevölkerung“ [2] im Leistungsbereich der Radioonkologie zu benennen. Grundsätzlich werden die gesetzlichen Anforderungen sowie die Anforderungen der Organzentren berücksichtigt. Im Versorgungslevel 3 werden zusätzlich zu den notwendigen onkologischen Netzwerkstrukturen komplexe Bestrahlungstechniken (u. a. Brachytherapie und Stereotaxie) und eine eigenständige stationäre radioonkologische Einrichtung als Indikator erhöhten Aufwands- und Vorhaltebedarfs beschrieben.

**Table 1:** 

	Level 2 (LG 18.1)	Level 3 (LG 18.2)
–	Station für Radioonkologie oder ausgewiesene Betten für Strahlentherapie (von Radioonkolog:in betreut)	Station für Radioonkologie
**Qualifikation ärztliche Leitung**	Facharzt/-ärztin für Strahlentherapie^a^	Facharzt/-ärztin für Strahlentherapie^a^
**Qualifikation pflegerische Leitung**	Examinierte Krankenpflegekraft^a^	Examinierte Krankenpflegekraft^a^
*–*	*Patient:in kann dort ambulant und/oder stationär behandelt werden, ohne dass behandelnder Arzt und Gerät gewechselt werden müssen*	*Patient:in kann dort ambulant und/oder stationär behandelt werden, ohne dass behandelnder Arzt und Gerät gewechselt werden müssen*
**Verfügbarkeit und Qualifikation von Ärzten**	*Strahlentherapeutische Einrichtung*: Ausstattung entsprechend Richtlinie Strahlenschutz in der Medizin (definiert pro Beschleuniger und Anzahl angewandter Techniken), insgesamt mind. 2 Fachärzt:innen für Strahlentherapie pro Organkrebszentrum (pro FA/FÄ maximal 3 Zentren) verfügbar^a^ *plus* 8/5 – 1 Facharzt/-ärztin für Strahlentherapie – 1 Arzt/Ärztin je 10 Betten 24/7 1 Facharzt/Fachärztin verfügbar, ggf. über Kooperation	*Strahlentherapeutische Einrichtung*: Ausstattung entsprechend Richtlinie Strahlenschutz in der Medizin (definiert pro Beschleuniger und Anzahl angewandter Techniken), insgesamt mind. 2 Fachärzt:innen für Strahlentherapie pro Organkrebszentrum (pro FA/FÄ maximal 3 Zentren) verfügbar^a^ *plus* mindestens 3 Ärzt:innen plus 8/5 – 1 Facharzt/Fachärztin für Strahlentherapie – 1 Arzt/Ärztin je 10 Betten 24/7 1 Facharzt/-ärztin verfügbar, 1 Arzt/Ärztin anwesend
**Weiterbildungsbefugnis Strahlentherapie**	Partielle Weiterbildung in der strahlentherapeutischen Einrichtung^1^ möglich	Volle Weiterbildung in der strahlentherapeutischen Einrichtung^1^ möglich
**Pflegerischer Stellenschlüssel**	Gemäß Pflegepersonaluntergrenzen-Verordnung	Gemäß Pflegepersonaluntergrenzen-Verordnung
**Anteil mit Fachweiterbildung**	1 onkologische Fachpflegekraft verfügbar am Krankenhausstandort^a^	1 onkologische Fachpflegekraft verfügbar am Krankenhausstandort^a^
**Physik**	*Werktäglich* 2 medizinische Physikexpert:innen entsprechend Richtlinie Strahlenschutz in der Medizin (definiert durch pro Beschleuniger und Anzahl angewandte Techniken)^a^	*Werktäglich* entsprechend Richtlinie Strahlenschutz in der Medizin (definiert durch pro Beschleuniger und Anzahl angewandte Techniken), mindestens 3 medizinische Physikexpert:innen
**MTR**	*Werktäglich* 2/Beschleuniger entsprechend Richtlinie Strahlenschutz in der Medizin (definiert durch pro Beschleuniger und Anzahl angewandte Techniken)^a^ plus 1/20 Betten (erhöhter Lagerungsaufwand)	*Werktäglich* 2/Beschleuniger entsprechend Richtlinie Strahlenschutz in der Medizin (definiert durch pro Beschleuniger und Anzahl angewandte Techniken)^a^ plus 1/20 Betten (erhöhter Lagerungsaufwand)
**Rufbereitschaft**	–	24/7 verfügbar – 1 Facharzt/-ärztin für Strahlentherapie – 1 Medizinphysikexpert:in
**Technische Vorhaltung**	Mindestens 1 Linearbeschleuniger plus Zugriff auf 2. Beschleuniger (Ausfallkonzept)^a^	2 Linearbeschleuniger am Standort
	Therapieplanung (3-D- und IMRT), virtuelle Simulation oder Therapiesimulator^a^	Therapieplanung (3-D- und IMRT), virtuelle Simulation oder Therapiesimulator^a^
	Planungs-CT^a^	Planungs-CT^a^
	Unmittelbare Anbindung Zentrallabor	Unmittelbare Anbindung Zentrallabor
	Anbindung Radiologie inkl. Zugriffsmöglichkeit auf Kernspintomographie^a^	Anbindung Radiologie inkl. MRT inkl. Zugriffsmöglichkeit auf Kernspintomographie^a^
	Anbindung Nuklearmedizin inkl. PET/CT je nach Anforderung Zertifikat des Organkrebszentrums^a^	Anbindung Nuklearmedizin inkl. PET/CT
	–	1 Brachytherapieeinrichtung (Afterloading-Technik)^1^
**Verfügbarkeit therapeutischer Verfahren**	*3‑D-konformale Strahlentherapie*^a^ IMRT^a^/VMAT oder vergleichbares Verfahren Strahlentherapie mit Berücksichtigung der Atembewegung Bildgeführte Strahlentherapie (IGRT)^a^ Palliative Strahlentherapie^a^	*3‑D-konformale Strahlentherapie*^a^ IMRT^a^/VMAT oder vergleichbares Verfahren Strahlentherapie mit Management der Atembewegung Bildgeführte Strahlentherapie (IGRT)^a^ Stereotaxie zerebraler Tumoren^a^ Palliative Strahlentherapie^a^
	*Mindestens eines der folgenden Verfahren*: Zerebrale stereotaktische Bestrahlung ein- oder mehrzeitig (8-523.02-04, 8‑523.12-14, 8‑523.2), extrazerebrale stereotaktische Bestrahlung ein- oder mehrzeitig (8-523.05-07, 8‑523.15-17, 8‑523.2), intraoperative Strahlentherapie (8-523.6, 8‑52d), Brachytherapie (8-524), Neuroachsenbestrahlung, adaptive Strahlentherapie, Rebestrahlung in kurativ intendiert vorbestrahltem Bereich (Qualitätsanforderungen: Rekonstruktion der Vorbestrahlung, biologische Dosisabschätzung Erst- und Rebestrahlung) Qualifikation wie empfohlen^b^ [15, 16]	*Mindestens drei der folgenden Verfahren:* Zerebrale stereotaktische Bestrahlung ein- oder mehrzeitig (8-523.02-04, 8‑523.12-14, 8‑523.2), extrazerebrale stereotaktische Bestrahlung ein- oder mehrzeitig (8-523.05-07, 8‑523.15-17, 8‑523.2), Ganzkörperbestrahlung (8-523.4), Ganzhautbestrahlung (8-523.5), intraoperative Strahlentherapie (8-523.6, 8‑52d), Brachytherapie interstitiell (8-525) und intrakavitär (8-524.0/8-524.2), Neuroachsenbestrahlung, Partikeltherapie (8-52a-c), adaptive Strahlentherapie mit dediziertem System, Rebestrahlung in kurativ intendiert vorbestrahltem Bereich (Qualitätsanforderungen: Rekonstruktion der Vorbestrahlung, biologische Dosisabschätzung Erst- und Rebestrahlung) oder OPS 8‑52 a‑c, 8–52 d, 8‑523.0, -523.1, -523.2, -523.4, -23.5, -523.6, -523.7, -524.0‑2 und 5‑6, -525.0‑2, -526, -52d Qualifikation wie empfohlen^b^ [15, 16]
	*Medikamentöse Tumortherapie inkl. Supportivtherapie in Verantwortung einer Fachärztin/eines Facharztes für Strahlentherapie (fakultativ)* Definition medikamentöse Tumortherapie: OPS 6‑00, 8‑54 bei ICD-10 C-Diagnose Qualifikation: mind. 50 systemische Tumortherapien in Kombination mit Bestrahlungen bei soliden Tumoren^a^	*Medikamentöse Tumortherapie inkl. Supportivtherapie in der strahlentherapeutischen Einrichtung*^*1*^* in Verantwortung einer Fachärztin/eines Facharztes für Strahlentherapie* Definition medikamentöse Tumortherapie: OPS 6‑00, 8‑54 bei ICD-10 C-Diagnose Qualifikation: mind. 50 systemische Tumortherapien in Kombination mit Bestrahlungen bei soliden Tumoren
**Qualitätsmanagement**	Prüfverfahren der Ärztlichen Stelle^a^ [10]	Prüfverfahren der Ärztlichen Stelle^a^ [10]
	Kooperationspartner in Organkrebszentren zertifiziert nach DKG^a^	Kooperationspartner in Organkrebszentren zertifiziert nach DKG^a^
	–	Teilnahme an klinischen Studien verpflichtend^1^
**Bauliche Struktur**	*Entsprechend Vorgaben der Strahlenschutzverordnung* Die räumliche Anordnung der stationären Betten muss geeignet sein, eine komplette Versorgung durch eine Fachärztin/einen Facharzt für Strahlentherapie zu ermöglichen. Eine Zuordnung strahlentherapeutischer Betten über mehrere Gebäude ist nicht zulässig	*Entsprechend Vorgaben der Strahlenschutzverordnung* Die räumliche Anordnung der stationären Betten muss geeignet sein, eine komplette Versorgung durch eine Fachärztin/einen Facharzt für Strahlentherapie zu ermöglichen. Eine Zuordnung strahlentherapeutischer Betten über mehrere Gebäude ist nicht zulässig
**Sonstiges**	*Tumordokumentation*^a^	*Tumordokumentation*^a^
	Schmerztherapie am Standort^a^	Schmerztherapie am Standort^a^
	*Palliativmedizin LG 16 oder Facharzt/Fachärztin mit Zusatzbezeichnung Palliativmedizin am Standort*^a^	*Palliativmedizin LG 16 oder Facharzt/Fachärztin mit Zusatzbezeichnung Palliativmedizin am Standort*^a^
	Ernährungsberatung am Standort^a^	Ernährungsberatung am Standort^a^
	Psychoonkologie (Liaisondienst) am Standort^a^	Psychoonkologie am Standort^a^
	*Sozialdienst am Standort*^a^	*Sozialdienst am Standort*^a^
	Physiotherapie am Standort^a^	Physiotherapie am Standort^a^ Logotherapie entsprechend Anforderung Organkrebszentrum am Standort^a^
	*Intensivmedizin LG 13.0*	*Intensivmedizin und Anästhesie LG 13.0*
	*Fort- und Weiterbildung*^a^ Es ist ein Qualifizierungsplan für das ärztliche, pflegerische und sonstige Personal vorzulegen, in dem die für einen Jahreszeitraum geplanten Qualifizierungen dargestellt sind	*Fort- und Weiterbildung*^a^ Es ist ein Qualifizierungsplan für das ärztliche, pflegerische und sonstige Personal vorzulegen, in dem die für einen Jahreszeitraum geplanten Qualifizierungen dargestellt sind
	*Nachweis der Anwendung der Qualitätsindikatoren der S3-Leitlinie entsprechend den Zertifikaten, für die die Radioonkologie Hauptkooperationspartner ist* (entsprechend Kennzahlenbogen: https://www.krebsgesellschaft.de/zertdokumente.html)^a^	*Nachweis der Anwendung der Qualitätsindikatoren der S3-Leitlinie entsprechend den Zertifikaten, für die die Radioonkologie Hauptkooperationspartner ist* (entsprechend Kennzahlenbogen: https://www.krebsgesellschaft.de/zertdokumente.html)^a^
	– Teilnahme an den mind. wöchentlich stattfindenden multidisziplinären Tumorkonferenzen entsprechend tumorspezifischen Zertifikaten^a^ – Teilnahme an MM-Konferenzen und Durchführung Qualitätszirkel (3×/J) entsprechend tumorspezifischen Zertifikaten^a^	– Teilnahme an den mind. wöchentlich stattfindenden multidisziplinären Tumorkonferenzen entsprechend tumorspezifischen Zertifikaten^a^ – Teilnahme an MM-Konferenzen und Durchführung Qualitätszirkel (3×/J) entsprechend tumorspezifischen Zertifikaten^a^
	Sprechstunden^a^ – Für jeden Pat. ist vor Beginn der Radiatio eine ärztliche Vorstellung sicherzustellen – Während einer Bestrahlungsserie ist mind. 1× ein dokumentierter ärztlicher Kontakt in der behandelnden Strahlentherapieeinrichtung sicherzustellen	Sprechstunden^a^ – Für jeden Pat. ist vor Beginn der Radiatio eine ärztliche Vorstellung sicherzustellen – Während einer Bestrahlungsserie ist mind. 1× ein dokumentierter ärztlicher Kontakt in der behandelnden Strahlentherapieeinrichtung sicherzustellen
	*Zuordnung zu bestehenden Leistungsgruppen* 1.3.1 Gastroenterologie; 1.4 Hämatologie und Onkologie; 1.8.1 Pneumologie; 2.4.1‑6 Orthopädie; 2.4.9 onkologische Orthopädie und Unfallchirurgie; 2.5.2 onkologische plastische und rekonstruktive Chirurgie; 2.6.1 onkologische Thoraxchirurgie; 2.7.2.1 onkologische Lebereingriffe; 2.7.3.1 onkologische Ösophagus‑/Mageneingriffe; 2.7.4.1 onkologische Pankreaseingriffe; 2.7.5.1 onkologische kolorektale Eingriffe; 2.7.6 weitere onkologische chirurgische Eingriffe; 3.1.1 Karzinome außer Mamma-Ca; 3.1.2 Senologie/Mamma-Ca; 4.2 Kinder- und Jugendhämatologie und -onkologie; 4.2.1 Kinderonkologie; 4.2.2 Kinderhämatologie und -onkologie-Stammzelltransplantationen; 5.2 komplexe Augenheilkunde; 6.3 Dermatoonkologie; 7.1.1 onkologische HNO; 8.1.1 onkologische MKG; 9.1.2 onkologische Neurochirurgie inkl. Stereotaxie; 11.1.1 onkologische Urologie; 16.1‑3 Palliativmedizin	

^a^Entsprechend Vorgaben Erhebungsbogen Radioonkologie DKG (EB Radio; https://www.krebsgesellschaft.de/zertdokumente.html)

^b^Guckenberger et al. [15], Schmitt et al. [16]

Die Matrix der Anforderungen an die Versorgungslevel ist ein Arbeitspapier auf der Basis der gesetzlichen Vorgaben, Empfehlungen der Strahlenschutzkommission, der onkologischen Qualitätssicherung (Zertifikate der DKG) sowie der „Erhebung zur Häufigkeit der Strahlentherapien in Deutschland“ des Bundesamts für Strahlenschutz [1]. Es ist zu betonen, dass es sich um ein vorläufiges Arbeitsergebnis handelt, das voraussichtlich im Laufe des Reformvorhabens weiterentwickelt wird.

## Zusammenfassung

Die DEGRO begrüßt das Konzept der Leistungsbereiche und Versorgungslevel ausdrücklich. Die qualitativ hochwertige radioonkologische Behandlung stationärer Patienten in Zentren und Spitzenzentren erfordert das Vorhalten und Instandhalten moderner hochspezialisierter Bestrahlungsgeräte sowie die personell ausreichende Ausstattung dieser Geräte mit qualifiziertem Fachpersonal. Um die hohe Qualität der Patientenversorgung zu bewahren und den hohen Ambulantisierungsgrad in der Radioonkologie zu erhalten, muss die Radioonkologie als eigener Leistungsbereich abgebildet werden.
